# Photosynthetic performance of rocket (*Eruca sativa*. Mill.) grown under different regimes of light intensity, quality, and photoperiod

**DOI:** 10.1371/journal.pone.0257745

**Published:** 2021-09-27

**Authors:** Naif Ali Elmardy, Ahmed F. Yousef, Kui Lin, Xiwen Zhang, Muhammad Moaaz Ali, Sobhi F. Lamlom, Hazem M. Kalaji, Katarzyna Kowalczyk, Yong Xu

**Affiliations:** 1 College of Horticulture, Fujian Agricultural and Forestry University, Fuzhou, China; 2 Department of Horticulture, College of Agriculture, University of Al-Azhar (Branch Assiut), Assiut, Egypt; 3 Institute of Machine Learning and Intelligent Science, Fujian University of Technology, Fuzhou, China; 4 Plant Production Department, Faculty of Agriculture Saba Basha, Alexandria University, Alexandria, Egypt; 5 Department of Plant Physiology, Institute of Biology, Warsaw University of Life Sciences SGGW, Warsaw, Poland; 6 Institute of Technology and Life Sciences, National Research Institute, Falenty, Raszyn, Poland; 7 Department of Vegetable and Medicinal Plants, Institute of Horticultural Sciences, Warsaw University of Life Sciences-SGGW, Warsaw, Poland; 8 College of Mechanical and Electronic Engineering, Fujian Agriculture and Forestry University, Fuzhou, China; Bangabandhu Sheikh Mujibur Rahman Agricultural University, BANGLADESH

## Abstract

In recent years, much effort has been devoted to understanding the response of plants to various light sources, largely due to advances in industry light-emitting diodes (LEDs). In this study, the effect of different light modes on rocket (*Eruca sativa*. Mill.) photosynthetic performance and other physiological traits was evaluated using an orthogonal design based on a combination between light intensity, quality, and photoperiod factors. Some morphological and biochemical parameters and photosynthetic efficiency of the plants were analyzed. Plants grew in a closed chamber where three light intensities (160, 190, and 220 μmol m^-2^ s^-1^) provided by LEDs with a combination of different ratios of red, green, and blue (R:G:B- 7:0:3, 3:0:7, and 5:2:3) and three different photoperiods (light/dark -10/14 h, 12/12 h, and 14/10 h) were used and compared with white fluorescent light (control). This experimental setup allowed us to study the effect of 9 light modes (LM) compared to white light. The analyzes performed showed that the highest levels of chlorophyll *a*, chlorophyll *b*, and carotenoids occurred under LM4, LM3, and LM1, respectively. Chlorophyll *a* fluorescence measurement showed that the best effective quantum yield of PSII photochemistry Y(II), non-photochemical quenching (NPQ), photochemical quenching coefficient (qP), and electron transport ratio (ETR) were obtained under LM2. The data showed that the application of R7:G0:B3 light mode with a shorter photoperiod than 14/10 h (light/dark), regardless of the light intensity used, resulted in a significant increase in growth as well as higher photosynthetic capacity of rocket plants. Since, a clear correlation between the studied traits under the applied light modes was not found, more features should be studied in future experiments.

## 1. Introduction

Rocket (*Eruca sativa*. Mill.) plant is a commercially important salad crop grown throughout the world. The leaves are known for their distinctive pungent flavor and are often consumed raw in salads [1–3].

Plants use light as an environmental signal and as a source for photosynthesis by responding to light intensity, quality, and duration [4]. Light is sensed by plant photosensors that include cryptochromes, phytochromes, and phototropin, and they output a variety of specific physiological responses through these sensors [5].

Traditionally, incandescent, fluorescent, and high-pressure sodium lamps with different spectral emissions have been used as substitutes of sunlight [6]. However, these types of artificial lights have certain limitations, such as high-power consumption, heat emission, and short life [7]. Recently, LED has been used as proper light source in the controlled agricultural environment because they have desirable characteristics such as low mass, safety, and durability [8–10].

It has been reported that different color ratios (different light spectrum) have a strong influence on plants’ physiological and developmental outcomes [11–14]. Other researches revealed the effect of light intensity on plant growth and development [13, 15–19], and photoperiod [13, 20]. However, little is known about the combined effect of light intensity, quality, and photoperiod on plants’ growth, development, and physiological response. In the present study, the effects of the aforementioned factors on of rocket (*Eruca sativa*. Mill.) were studied. The experiment was based on 3 factors with 3 levels L9 (3^3^) as it is shown in Table 1 (an orthogonal experimental design method).

**Table 1:** The properties of treatments used in the study.

Treatments	Photon flux density (μmol m^-2^ s^-1^)	Light spectral ratios	Photoperiod Light/Dark (h)	Peak wavelength λp (nm)	Layout of the L9 (3^3^) matrix		
					A	B	C
**LM1**	220	R7:G0:B3	10/14	660:460	1	1	1
**LM2**	220	R3:G0:B7	12/12	660:460	1	2	2
**LM3**	220	R5:G2:B3	14/10	660:530:460	1	3	3
**LM4**	190	R7:G0:B3	12/12	660:460	2	1	2
**LM5**	190	R3:G0:B7	14/10	660:460	2	2	3
**LM6**	190	R5:G2:B3	10/14	660:530:460	2	3	1
**LM7**	160	R7:G0:B3	14/10	660:460	3	1	3
**LM8**	160	R3:G0:B7	10/14	660:460	3	2	1
**LM9**	160	R5:G2:B3	12/12	660:530:460	3	3	2
**CK**	190	White fluorescent light	12/12	544	------	------	------

The aim of this study was to find out the optimal effect of a combination of light intensity, quality and photoperiod on the growth, photosynthetic pigments and performance, and biochemical and physiological properties of rocket plants. It was also aimed at revealing the mechanisms behind the different responses of the plants to the factors studied.

## 2. Material and methods

### 2.1. Growth conditions and LEDs light

**Fig 1:**
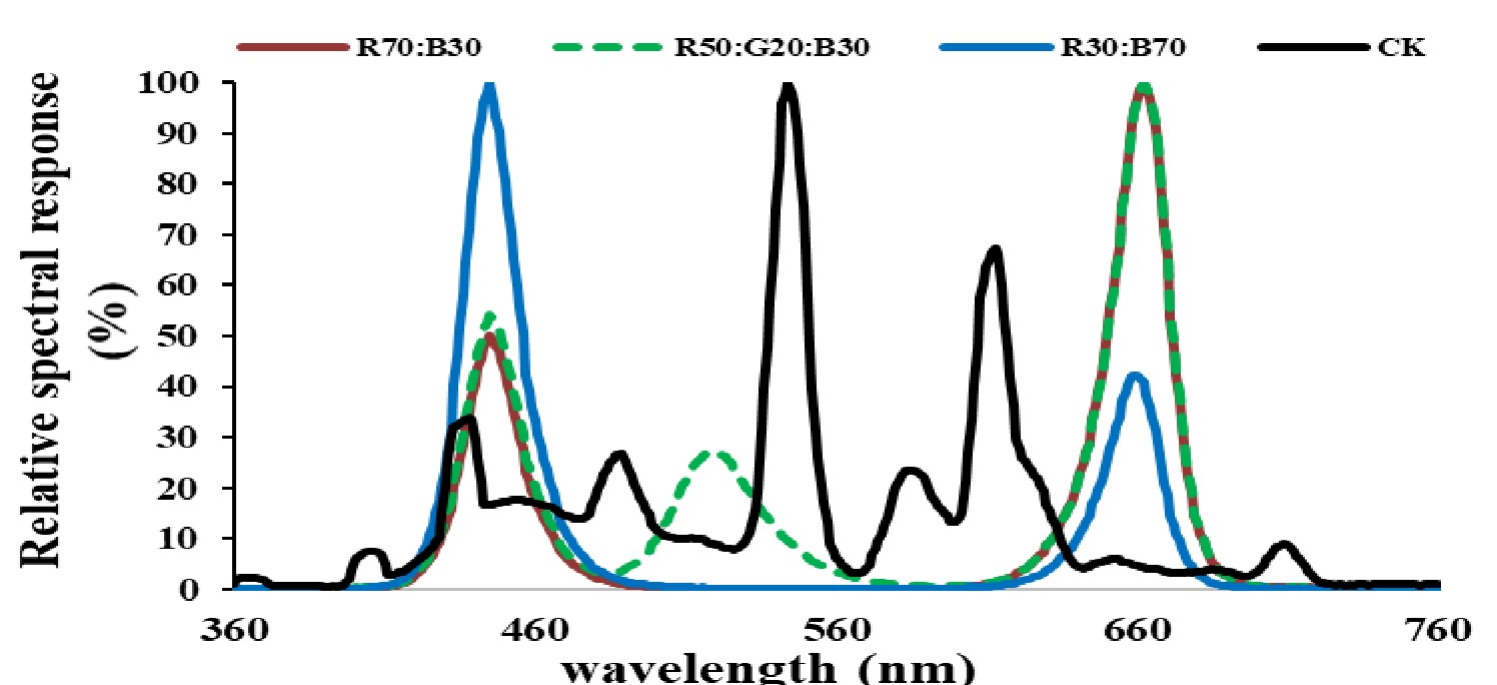
Relative spectral response of the light modes and control in the experiment.

The experiment was conducted in closed and equipped with LED light chambers at Fujian Agriculture and Forestry University, Fuzhou, China. The experimental system included 10 chambers; each had a dimension of 60 × 60 × 60 cm. The properties of growth conditions and LEDs light are shown in Table 1 and Fig 1.

### 2.2. Multiple factor experiment design

A multiple-factor experimental regular fractional design was used. Nine light modes (LM1– LM9) were applied (Table 1):

The intensities of LEDs light averaged over the plant growing period as A1- A3: 220, 190, and 160 μmol m^-2^ s^-1^.

The light spectrum is generated by different ratios of red, green, and blue (R:G:B) as B1-B3: = 7:0:3, 3:0:7, and 5:2:3. White fluorescent light of 190 μmol m^-2^ s^-1^ (mean value of the threes used intensities- 160, 190 and 220) was considered as reference/control (CK).

The light period during day/night as C1-C3: 10/14 h, 12/12 h, and 14/10 h.

### 2.3. Plant materials

The experimental system includes ten (10) treatments; each treatment has 3 chambers (same conditions) as replicates; each chamber has 60 × 60 × 60 cm dimensions. Rocket seeds were sown in pots (W 10 cm × H 12 cm, Luoxi Plastic Products Co., Shandong, China) that were filled with the commercial growing substrate (N1:P1:K1 ≥ 3%, Organic matter ≥ 45%, pH 5.5–6.5, Jiangping Enterprise Co., Fujian, China). In total, 20 pots were sown in each growth chamber, 10 plants were sown in each pot, where CO_2_ concentration level was 580–600 mg L^-1^, and the relative humidity was maintained at 70 ± 10% and a temperature of 25°C ± 2 throughout the day. Irrigation was provided for the seedlings as required. The pots were watered according to the plants’ needs by observing the moisture of rooting media. Hoagland’s solution (0.4 NH_4_H_2_PO_4_; 2.4 KNO_3_; 1.6 Ca(NO_3_)_2_; 0.8 MgSO_4_; 0.1 Fe as Fe-chelate; 0.023 B as B(OH)_3_ (boric acid); 0.0045 Mn as MnCl_2_; 0.0003 Cu as CuCl_2_; 0.0015 Zn as ZnCl_2_; 0.0001 Mo as MoO_3_ or (NH_4_)6Mo7O_24_; Cl as chlorides of Mn, Zn, and Cu (all concentrations in units of μM L^-1^)) was used for plant fertigation.

### 2.4. Growth and development parameters

Growth parameters were estimated on 28^th^ days after sowing; values are means of four replicates, 6 plants in each replicate. Measurements of plant height, hypocotyl length, and root length were done manually (cm). Stem diameter was measured using digital calipers (mm). Total leaf area (cm^2^) (summation of leaf areas) was estimated as described by Pandey and Singh [21]. Dry weight content % was estimated using the equation (plant fresh weight) * (1- percentage of moisture of plant), where the fresh and dry mass was weighed using an electronic balance (0.0001 g).

### 2.5. Photosynthetic pigments content

Chlorophyll contents were determined spectrophotometrically from fresh leaves of different 6 plants in each treatment 28 days after sowing. Fresh leaves tissue (0.2 g) were cut, ground well then put in 5 mL 95% ethanol and filtered and the volume was made up to 25 mL using 95% ethanol and calculated and the Knight and Mitchell [22] formulas were used to determine the chlorophyll contents: Chl *a* (mg g^-1^) = (13.95OD_665_- 6.88OD_649_)V/200 W; Chl *b* (mg g^-1^) = (24.96OD_649_−7.32OD_663_)V/200W; C (mg g^-1^) = (1000OD_470_-2.05Chl a-114.80Chl b) V/(245 × 200 W). Where (Chl *a*) = chlorophyll *a*, (Chl *b*) = chlorophyll *b*, (C) = carotenoid, mg/g; (V) = volume (25 mL) and (W) = sample weight (g).

### 2.6. Biochemical compounds content

Fresh leaves were chopped into small pieces to measure some chosen biochemical compounds, and fresh samples weighed (0.5, 0.2, 2, and 0.5 g) for protein, sugar, Vitamin C, and nitrate content, respectively. The soluble protein content was evaluated using the coomassie brilliant blue G250 method [23], while soluble sugar content was evaluated using the anthrone colorimetric method [24]. Wang and Huang [25] and Cataldo, Maroon [26] methods were used to measuring the Vitamin C content and nitrate content, respectively. The absorbance of the solution extracted was estimated at 630 nm (OD630), 595 nm (OD595), 500 nm (OD500), and 410 nm (OD410), respectively, and using a UV-5100B spectrophotometer (Unico, Shanghai, China). The biochemical contents were expressed using the following equations: Soluble protein content (mg g^-1^) = (C × VT)/(VS × W × 1000); Soluble sugar content (%) = (C/Vs × Vt)/(W × 106) × 100; Vitamin C content (mg g^-1^) = (C × Vt × 100)/(W × Vs); Nitrate content (mg kg^−1^ FW) = (C × Vt)/(W × Vs); Where C = protein/sugar/Vitamin C/nitrate content value from the standard curve, Vt = total volume of samples extracted (mL), Vs = taken sample solution (mL), and W = leaf fresh weight (g).

### 2.7. Plants’ photosynthetic efficiency

Chlorophyll *a* fluorescence measurements were made using a PAM 2500 Chlorophyll Fluorescence Meter (Heinz Walz GmbH, Effeltrich, Germany). The fourth leaf from each plant was selected randomly for this evaluation. The leaf area of the standard measuring head is 1.3 cm^2^ with atmospheric CO_2_ concentrations and a saturation pulse from red LEDs (8000 μmol m^-2^ s^-1^, 300 ms duration) were applied to determine the maximum chlorophyll fluorescence with closed PSII centers after dark-acclimation (Fm) and during illumination (Fm’). Fluorescence induction kinetics (first protocol) was measured after dark-acclimation for 30 min, then the rapid light curves (RLCs) (second protocol) were recorded immediately. The steady-state fluorescence was measured after 20 seconds of exposure to actinic light, similar to the mean value of the growth irradiance (190 μmol m^-2^ s^-1^). The light intensity gradient of the RLC was: 0, 2, 6, 64, 101, 198, 363, 619, 785, and 1160 μmol m^-2^ s^-1^.

Under dark and light acclimations, the effective quantum yield of PSII photochemistry Y(II) [27], the quantum yield of non-regulatory energy dissipation [Y(NO) = Fs/Fm], the quantum yield of regulatory energy dissipation [Y(NPQ) = 1-Y(II)-Y(NO)], non-photochemical quenching (NPQ), photochemical quenching (qP), and the electron transport rate (ETR) were measured. Measurements were conducted with 4 replicates per treatment (4 replicates × 4 leaves = 16 reads).

### 2.8. Statistical analysis

The Orthogonal Experimental design method was used to determine the number of experiments to be conducted. All the data were subjected to one-way analysis of variance (ANOVA). Duncan’s multiple range tests [28] was used to test the significant difference between the means at 0.05 significance level using SPSS software (Version 16 SPSS Inc. Chicago, Illinois). The importance of the three factors for the measured parameters was assessed according to the effectiveness of each factor [29] by the range value (*R*) using Excel 365 (v16.0). The most important impact factor has the greatest R-value. Adobe Illustrator software package version 23.0.3 was used to improve the quality of the images.

## 3. Results

### 3.1. Growth parameters

**Fig 2:**
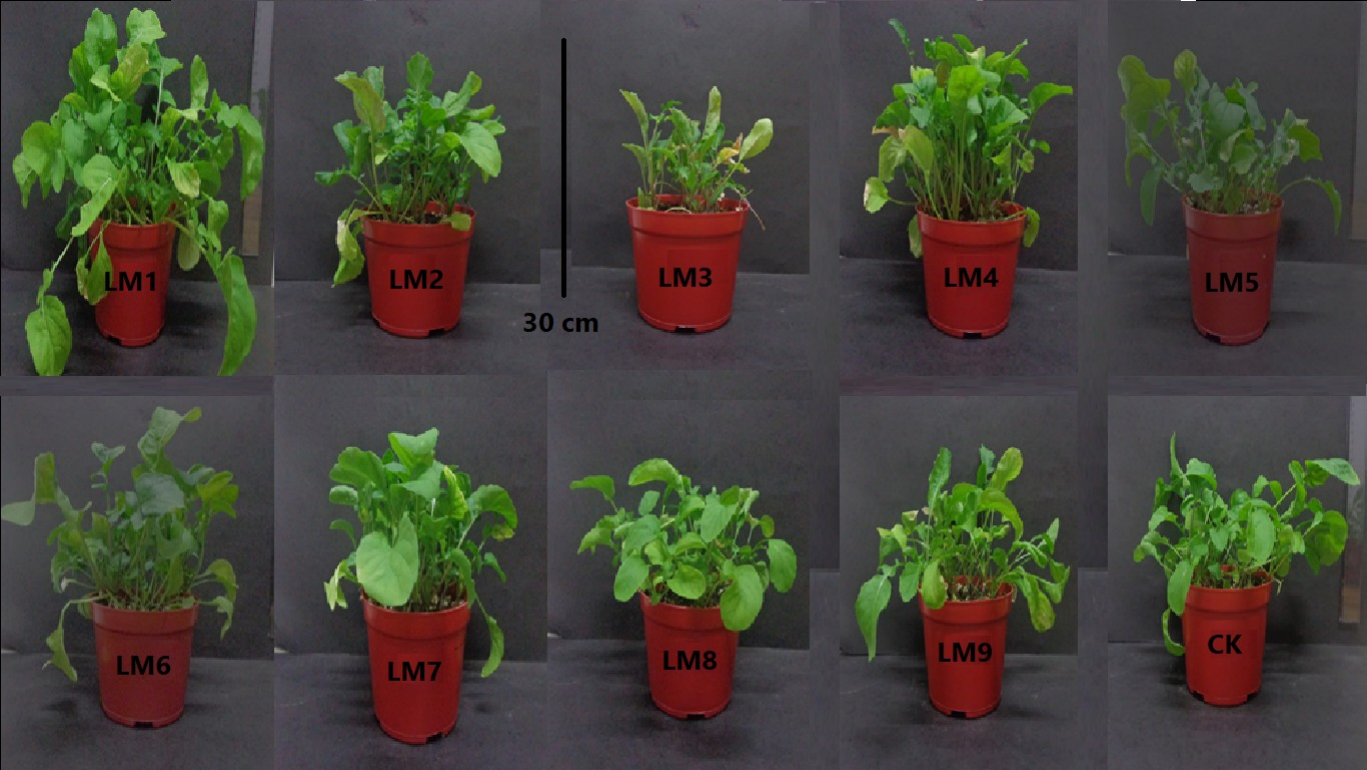
Rocket plants growth under different modes of LED lighting. For more details about the used light modes (LM), please refer to Table 1.

**Fig 3:**
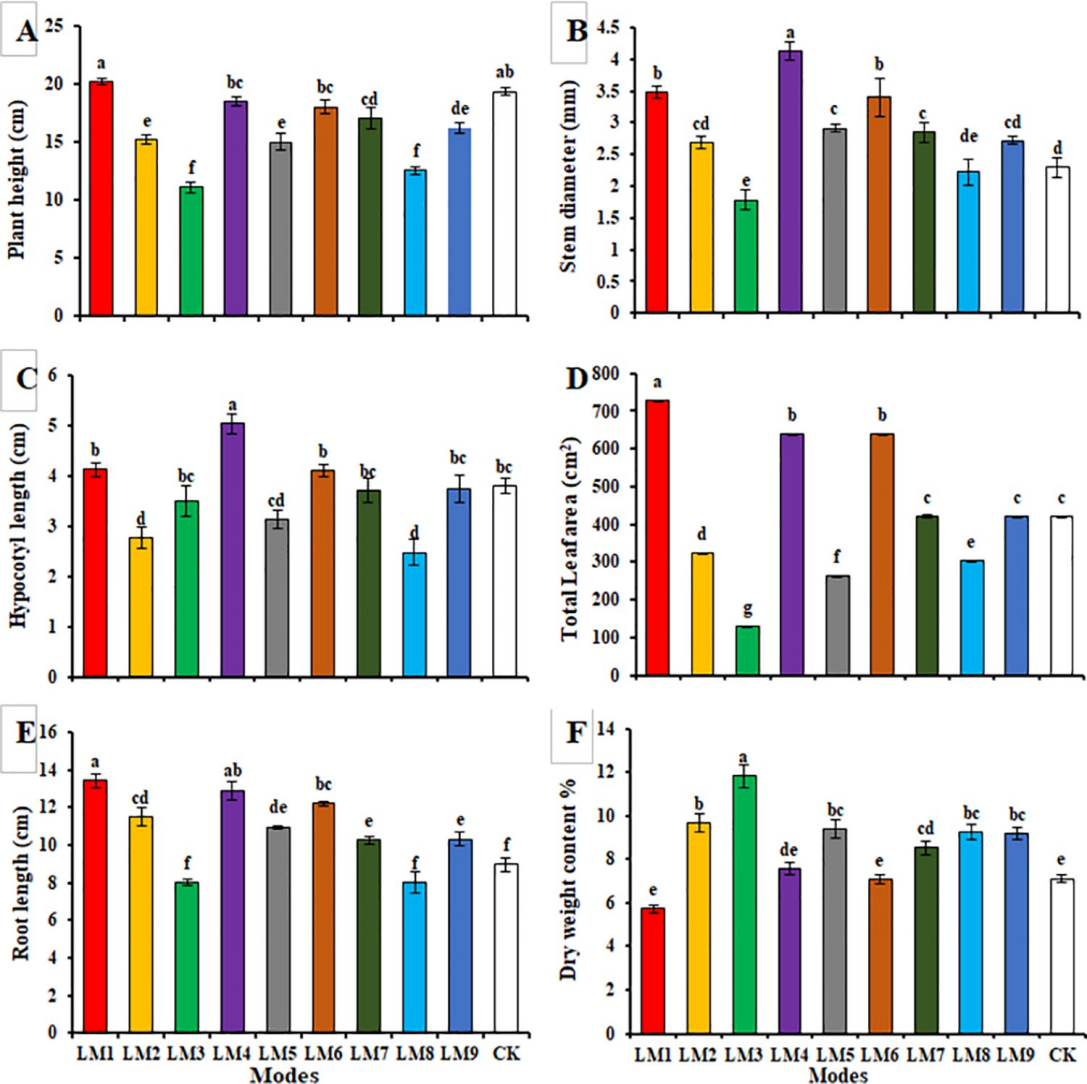
Effects of different lighting modes (LM) on the growth parameters of *E*. *Sativa*; plant height (A), stem diameter (B), hypocotyl length (C), total leaf area (D), root length (E), and dry weight content (F). Values are means of four replicates ± SE. Different letters between columns indicate significant differences according to Duncan’s multiple range test at P ≤ 0.05. LM1 = A1:B1:C1, LM2 = A1:B2:C2, LM3 = A1:B3:C3, LM4 = A2:B1:C2, LM5 = A2:B2:C3, LM6 = A2:B3:C1, LM7 = A3:B1:C3, LM8 = A3:B2:C1, LM9 = A3:B3:C1, CK = White fluorescent light.

The application of different lighting modes (LM) caused significant influence on the morphological appearances (Figs 2 and 3). Plant height, total leaf area, and root length were significantly highest under LM1 treatment, while the lowest plant height, total leaf area and root length were observed under LM8, LM3 and LM8 (Fig 3A, 3D, and 3E), respectively. The stem diameter of plants irradiated with LM4 (4.13 mm) was significantly larger than the other light modes (Fig 3B). Hypocotyl length of plants irradiated with LM4 (5.03 mm) was significantly higher than the other light modes, with LM8 having the lowest value (Fig 3C). Dry weight content of plants under LM3 (11.83%) and LM2 (9.68%) treatments was greater than the other light modes, while LM1 treatment had the lowest dry weight % content (5.72%) (Fig 3F).

On the other hand, according to the R-values, the order of influence of the three factors on growth characteristics of rocket plantss was observed in this study by using the orthogonal array design (Table 2). Table 2 shows that the order of impact of the three factors on plant height, stem diameter, hypocotyl length, total leaf area, rRoot length, and dry weight content was (B > C > A), (A > B > C), (B > A > C), (B > C > A), (A > B > C), (C > B > A), respectively.

**Table 2:** Results of the range and ANOVA of the L9 (33) matrix for the influence of combined, intensities of LEDs light (A), light spectral ratios (B), and photoperiod (C) on growth characteristics of rocket plants.

Parameters		Factors			ELF	BCm
		A	B	C		
**Plant height**	*R* value	1.91	4.33	2.53	B > C > A	A_1_B_1_C_1_
	*P* value	0.1805	0.0019	0.0599		
**Stem diameter**	*R* value	0.89	0.88	0.66	A > B > C	A_2_B_1_C_2_
	*P* value	< 0.0001	< 0.0001	< 0.0001		
**Hypocotyl length**	*R* value	0.78	1.50	0.40	B > A > C	A_2_B_1_C_2_
	*P* value	< 0.0001	< 0.0001	0.0013		
**Total leaf area**	*R* value	132.17	298.90	284.41	B > C > A	A_1_B_1_C_1_
	*P* value	0.0005	< 0.0001	< 0.0001		
**Root length**	*R* value	2.50	2.05	1.81	A > B > C	A_1_B_1_C_1_
	*P* value	0.0005	0.0009	0.0053		
**Dry weight content**	*R* value	1.07	2.16	2.56	C > B > A	A_1_B_3_B_3_
	*P* value	0.0242	< 0.0001	< 0.0001		

Range value (R)–the range of difference between the maximum and minimum average; (P-value)–ANOVA analysis of variance; ELF–The most influential level factors on the parameter gradually; BCm–The best level combination for each parameter.

Based on the average of growth characteristics derived from three factors at each level, the best combination of different factors with the levels to get the highest plant height, total leaf area, and root length was A_1_B_1_C_1_, which indicated that the maximum of these parameters presented at the intensity of light (220 μmol m^-2^ s^-1^), the ratio of (R7: G0: B3), and photoperiod (10 h/14 h). While the best combination of different factors with the levels to get the highest stem diameter and hypocotyl length was A_2_B_1_C_2_, which indicated that the maximum of these parameters presented at the intensity of light (190 μmol m^-2^ s^-1^), the ratio of (R7:G0:B3), and photoperiod (12 h/12 h).

ANOVA (Table 2) showed that these three factors were significant effects on growth performance parameters of rocket plants (*p* < 0.05), excepted factor A and C on plant height had no significant effects.

### 3.2. Chlorophyll and carotenoid contents

**Fig 4:**
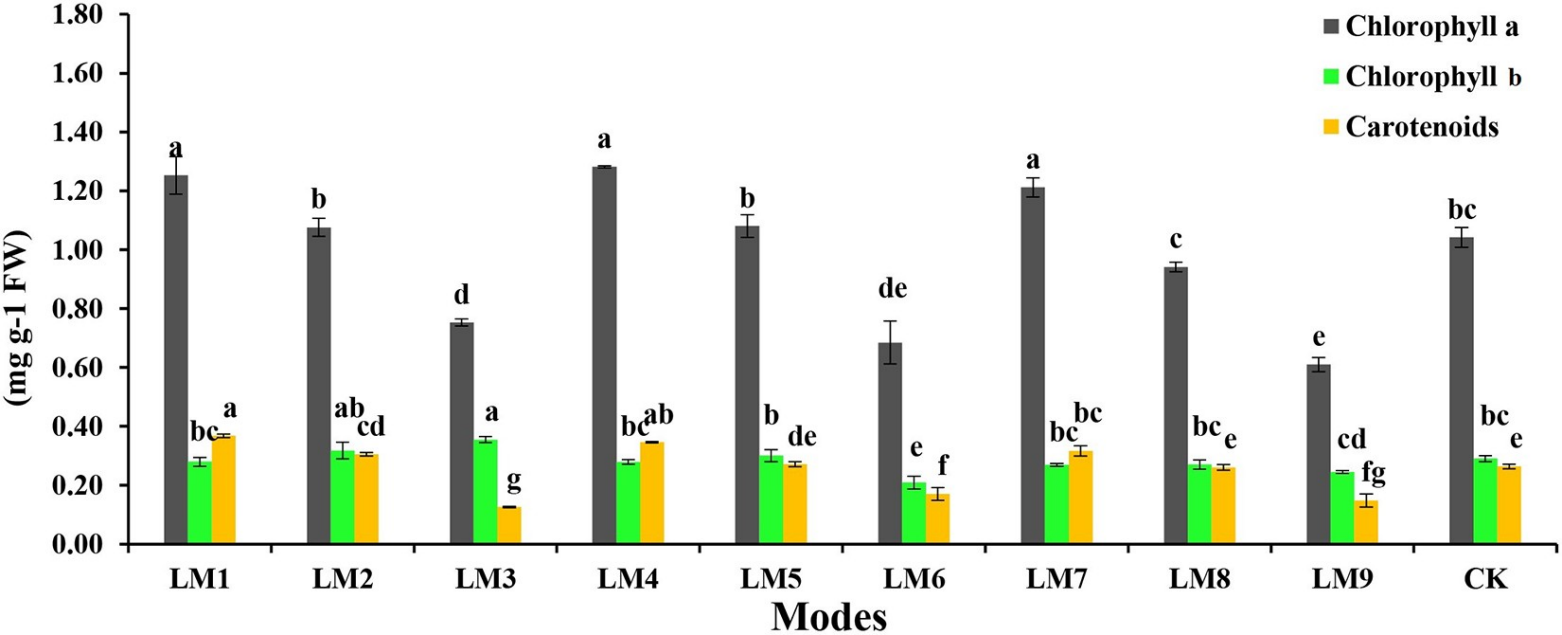
Effects of different lighting modes (LM) on chlorophyll *a*, chlorophyll *b*, and carotenoid content in leaves of *E*. *Sativa* plants. Values are means of four replicates ± SE. Different letters between columns indicate significant differences according to Duncan’s multiple range test at P ≤ 0.05. LM1 = A1:B1:C1, LM2 = A1:B2:C2, LM3 = A1:B3:C3, LM4 = A2:B1:C2, LM5 = A2:B2:C3, LM6 = A2:B3:C1, LM7 = A3:B1:C3, LM8 = A3:B2:C1, LM9 = A3:B3:C1, CK = White fluorescent light.

Chlorophyll and carotenoids contents in the leaves of rocket salad showed significant differences under changed LED treatments (Fig 4). In comparison with WFL treatment, Chl *a* content of rocket salad growing under LM1, LM4, and LM7 was higher than the other treatments. The Chl *b* content under LM3 showed a significant difference as compared to other treatments, except LM2. Plants treatment with LM6 caused the observation of the lowest Chl *b* content. The carotenoid content under LM1 was higher than that of other treatments, while the plants under LM3 treatment showed the lowest carotenoid content.

### 3.3. Biochemical compounds contents

**Fig 5:**
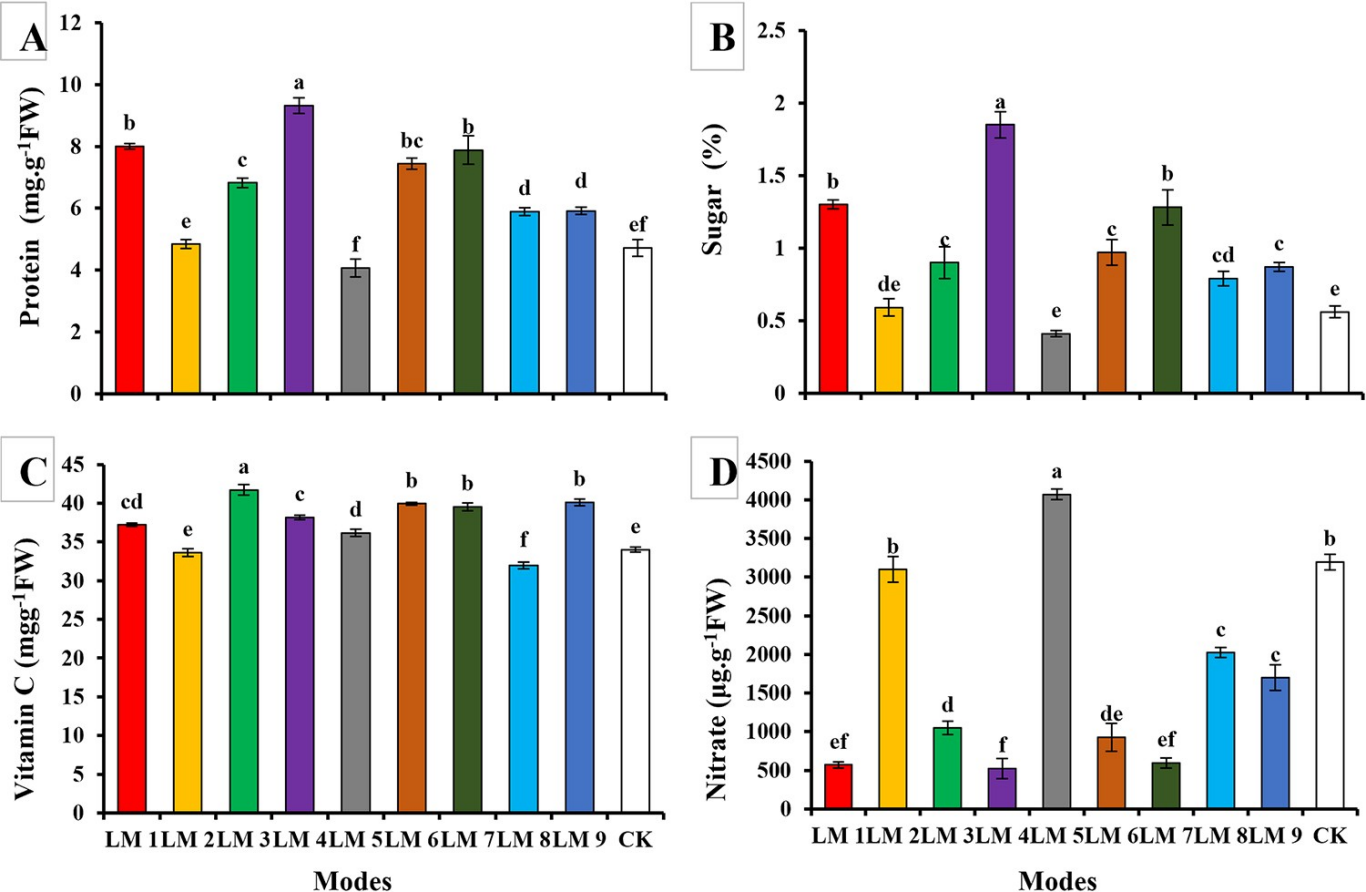
Effects of different lighting modes (LM) on protein (A), sugar (B), Vitamin C (C), and nitrate contents (D) in leaves of *E*. *Sativa* plants. Values are means of four replicates ± SE. Different letters between columns indicate significant differences according to Duncan’s multiple range test at P ≤ 0.05. LM1 = A1:B1:C1, LM2 = A1:B2:C2, LM3 = A1:B3:C3, LM4 = A2:B1:C2, LM5 = A2:B2:C3, LM6 = A2:B3:C1, LM7 = A3:B1:C3, LM8 = A3:B2:C1, LM9 = A3:B3:C1, CK = White fluorescent light.

Analysis of biochemical compounds content in the leaves of rocket (*E*. *Sativa*) plants showed that there were a significant difference depending on the applied light mode (Fig 5).

The effect of different light modes on *E*. *Sativa* plant showed that plants treated with LM5 and LM2 had the lowest protein content and were not significantly different from CK, while the highest protein content found under LM4 followed by LM1 and LM6 (Fig 5A).

Sugar content increased and was significantly affected by increasing the red-light ratio. The highest value of sugar content was recorded under light ratio (LM4), followed by (LM1) and (LM7), respectively. In contrast, plants grown under the ratio (LM5) had the lowest sugar content compared to CK (Fig 5B).

Plants grown under R5:G2:B3 light ratio showed a significantly increased vitamin C, especially under LM3, where its content was the highest among all the treatments, followed by LM6, LM7, and LM9 treatments. Plants treated with the same light ratio and photoperiod 14 h light/10 h dark showed the best values for vitamin C content. LM5 treatment caused a higher increment of vitamin C, as compared to LM2 and LM8 (Fig 5C).

The exact opposite was observed in the case of soluble nitrate content. The latter increased significantly and was affected by the decrease in red light ratio. Compared to the control, plants grown under LM5 R3:G0:B7 showed the highest value of soluble nitrate content in *E*. *Sativa* plants, while the lowest content was denoted under LM4 R7:G0:B3 light ratio (Fig 5D).

On the other hand, according to the R-values, the order of influence of the three factors on chlorophyll and biochemical contents of rocket plants was observed in this study by using the orthogonal array design (Table 3). Table 3 shows that the order of impact of the three factors on chlorophyll *a*, chlorophyll *b*, carotenoid, protein, sugar, vitamin C, and nitrate content was (B > A > C), (C > A > B), (B > C > A), (B > C > A), (B > C > A), (B > C > A), and (C > A > B), respectively.

**Table 3:** Results of the range and ANOVA of the L9 (3^3^) matrix for the influence of combined intensities of LEDs light (A), light spectral ratios (B), and photoperiod (C) on chlorophyll and biochemical contents of rocket plants.

Parameters		Factors			ELF	BCm
		A	B	C		
**Chlorophyll *a***	*R* value	0.11	0.57	0.06	B > A > C	A_2_B_1_C_2_
	*P* value	< 0.0001	< 0.0001	< 0.0001		
**Chlorophyll *b***	*R* value	0.056	0.026	0.06	C > A > B	A_1_B_3_C_3_
	*P* value	< 0.0001	0.0090	< 0.0001		
**Carotenoid**	*R* value	0.025	0.195	0.028	B > C > A	A_1_B_1_C_1_
	*P* value	0.0007	0.0848	0.0026		
**Protein**	*R* value	0.38	3.47	0.86	B > C > A	A_2_B_1_C_2_
	*P* value	0.4649	< 0.0001	0.0769		
**Sugar**	*R* value	0.15	0.88	0.24	B > C > A	A_2_B_2_C_3_
	*P* value	0.3299	< 0.0001	0.0667		
**Vitamin C**	*R* value	0.90	6.67	2.78	B > C > A	A_1_B_3_C_3_
	*P* value	0.0190	< 0.0001	< 0.0001		
**Nitrate content**	*R* value	402.40	2501.60	730.13	C > A > B	A_2_B_2_C_3_
	*P* value	0.2002	< 0.0001	0.0082		

Range value (R)–the range of difference between the maximum and minimum average; (P-value)–ANOVA analysis of variance; ELF–The most influential level factors on the parameter gradually; BCm–The best level combination for each parameter.

Based on the average of chlorophyll and biochemical contents derived from three factors at each level, the A_2_B_1_C_2_ was the best combinations gave the highest chlorophyll *a*, and protein, which indicated that the maximum of these parameters presented at (intensity 190 μmol m^-2^ s^-1^ + ratio (R7:G0:B3) + photoperiod 12 h/12 h). While the best combination of different factors with the levels for the highest chlorophyll *b* and vitamin C was A_1_B_3_C_3_, which indicated that the maximum of these parameters presented with (intensity 220 μmol m^-2^ s^-1^ + ratio (R5:G2:B3) + photoperiod 14 h/10 h). The best combination of different factors with the levels for the highest chlorophyll *b* and vitamin C was A_2_B_2_C_3_, which indicated that the maximum of these parameters presented with (intensity 190 μmol m^-2^ s^-1^ + ratio (R3:G0:B7) + photoperiod 14 h/10 h).

ANOVA (Table 3) showed that these three factors significantly affected on chlorophyll and biochemical contents of rocket plants (*p* < 0.05), excepted factor B on carotenoid, factor A on nitrate content, and factor A and C on protein and sugar had no significant effects.

### 3.4. Chlorophyll *a* fluorescence measurements of dark-acclimated samples

**Fig 6:**
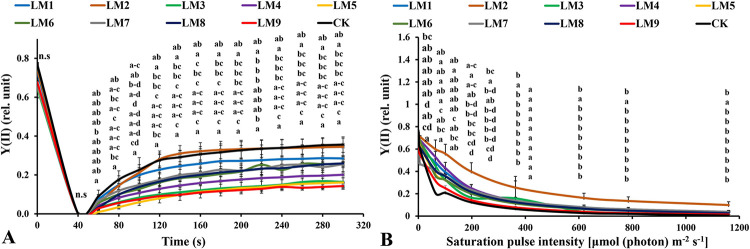
Effects of LED light modes on chlorophyll *a* fluorescence induction kinetics of the dark-acclimated (A) and RLC of the light-acclimated (B) of effective quantum yield of PSII photochemistry Y(II) in rocket leaves. The points are means ± SE of 4 replicates followed by the same letters meaning no different significantly according to the Duncan test (P ≤ 0.05). Sort significance letters from top to bottom according to the treatments (ML1-CK). LM1 = A1:B1:C1, LM2 = A1:B2:C2, LM3 = A1:B3:C3, LM4 = A2:B1:C2, LM5 = A2:B2:C3, LM6 = A2:B3:C1, LM7 = A3:B1:C3, LM8 = A3:B2:C1, LM9 = A3:B3:C1, CK = White fluorescent light.

**Fig 7:**
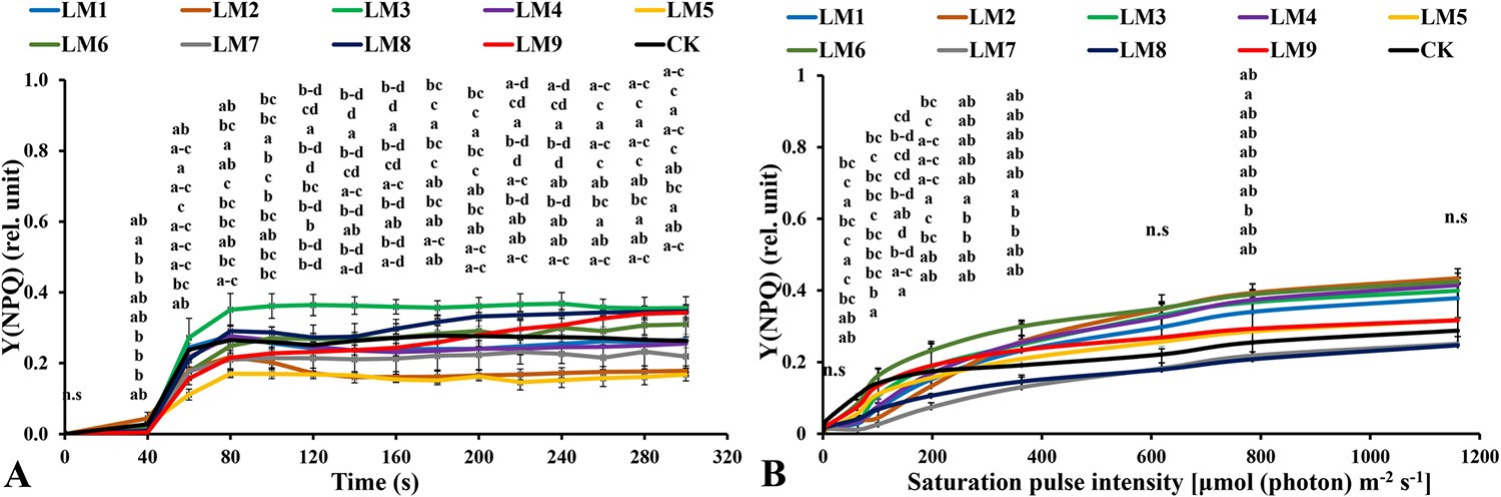
Effects of LED light modes on chlorophyll *a* fluorescence induction kinetics of the dark-acclimated (A) and RLC of the light-acclimated (B) of quantum yield of regulatory energy dissipation in PSII Y(NPQ) in rocket leaves. The points are means ± SE of 4 replicates followed by the same letters meaning no different significantly according to the Duncan test (P ≤ 0.05). Sort significance letters from top to bottom according to the treatments (ML1-CK). LM1 = A1:B1:C1, LM2 = A1:B2:C2, LM3 = A1:B3:C3, LM4 = A2:B1:C2, LM5 = A2:B2:C3, LM6 = A2:B3:C1, LM7 = A3:B1:C3, LM8 = A3:B2:C1, LM9 = A3:B3:C1, CK = White fluorescent light.

**Fig 8:**
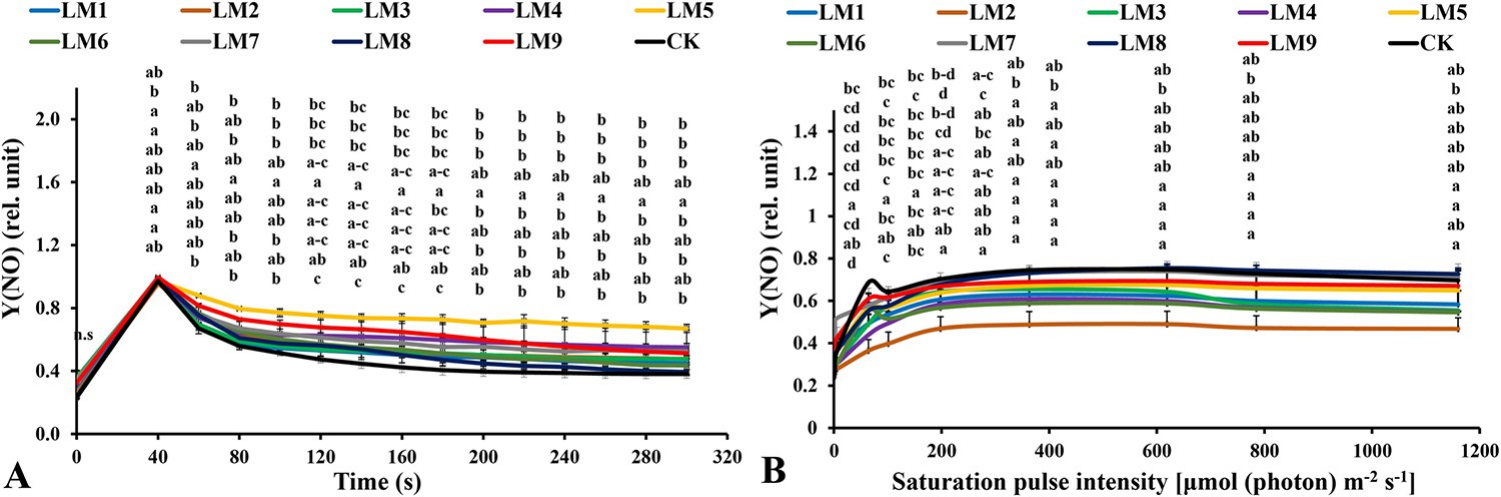
Effects of LED light modes on chlorophyll *a* fluorescence induction kinetics of the dark-acclimated (A) and RLC of the light-acclimated (B) of quantum yield of non-regulated energy dissipation in PSII Y(NO) in rocket leaves. The points are means ± SE of 4 replicates followed by the same letters meaning no different significantly according to the Duncan test (P ≤ 0.05). Sort significance letters from top to bottom according to the treatments (ML1-CK). LM1 = A1:B1:C1, LM2 = A1:B2:C2, LM3 = A1:B3:C3, LM4 = A2:B1:C2, LM5 = A2:B2:C3, LM6 = A2:B3:C1, LM7 = A3:B1:C3, LM8 = A3:B2:C1, LM9 = A3:B3:C1, CK = White fluorescent light.

Rapid light curves (RLCs) of dark-acclimated plants showed that the effective quantum yield of PSII photochemistry [Y(II)] increased rapidly with continued light exposure at all time points. The Y(II) of CK was significantly higher in plants at 60–100 and 240–300 seconds (Fig 6A), whereas the Y(II) of plants grown under LM2 was significantly higher at 120–220 seconds. The quantum yield of the regulated energy dissipation in PSII [Y(NPQ)] increased rapidly at all time points. The [Y(NPQ)] was highest in plants cultivated under LM3 treatment at 60–300 seconds (Fig 7A). The quantum yield of non-regulated energy dissipation in PSII [Y(NO)] increased rapidly at initial light exposure and decreased directly at 40 seconds with time elapsing. The [Y(NO)] was highest in plants treated with LM5 light mode at 60–300 seconds (Fig 8A).

**Fig 9:**
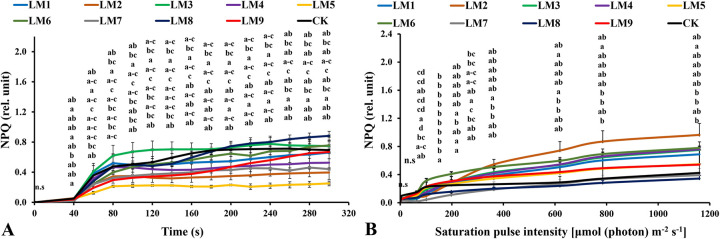
Effects of LED light modes on chlorophyll *a* fluorescence induction kinetics of the dark-acclimated (A) and RLC of the light-acclimated (B) of non-photochemical quenching (NPQ) in rocket leaves. The points are means ± SE of 4 replicates followed by the same letters meaning no different significantly according to the Duncan test (P ≤ 0.05). Sort significance letters from top to bottom according to the treatments (ML1-CK). LM1 = A1:B1:C1, LM2 = A1:B2:C2, LM3 = A1:B3:C3, LM4 = A2:B1:C2, LM5 = A2:B2:C3, LM6 = A2:B3:C1, LM7 = A3:B1:C3, LM8 = A3:B2:C1, LM9 = A3:B3:C1, CK = White fluorescent light.

**Fig 10:**
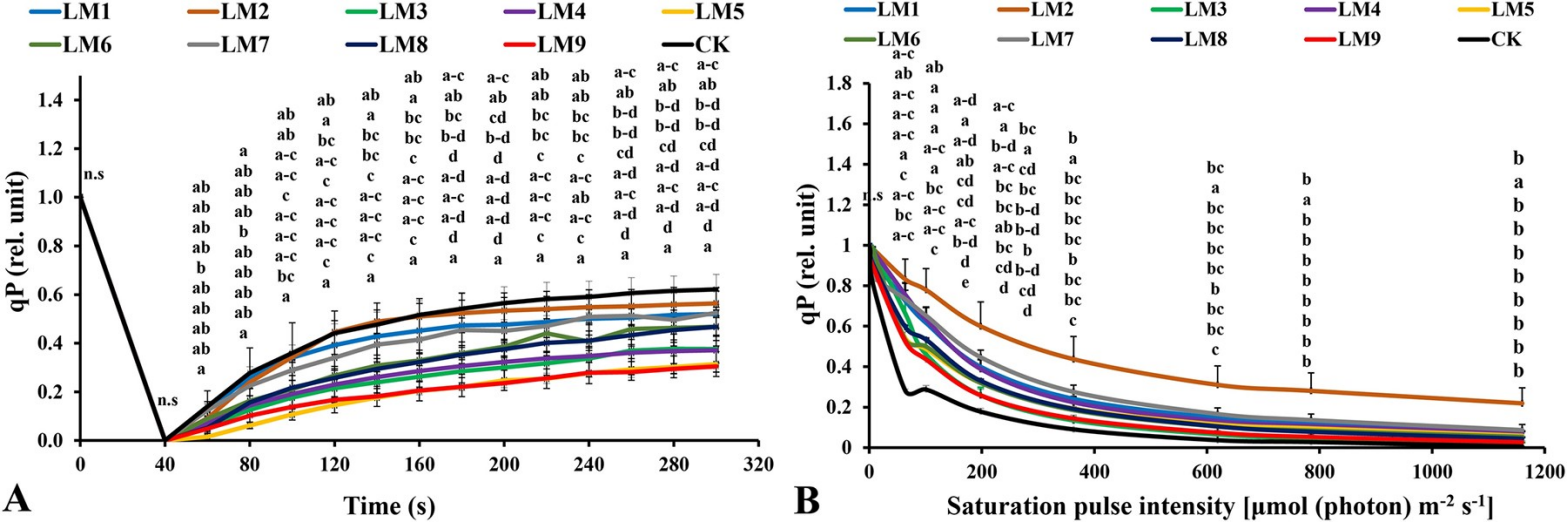
Effects of LED light modes on chlorophyll *a* fluorescence induction kinetics of the dark-acclimated (A) and RLC of the light-acclimated (B) of photochemical quenching coefficient (qP) in rocket leaves. The points are means ± SE of 4 replicates followed by the same letters meaning no different significantly according to the Duncan test (P ≤ 0.05). Sort significance letters from top to bottom according to the treatments (ML1-CK). LM1 = A1:B1:C1, LM2 = A1:B2:C2, LM3 = A1:B3:C3, LM4 = A2:B1:C2, LM5 = A2:B2:C3, LM6 = A2:B3:C1, LM7 = A3:B1:C3, LM8 = A3:B2:C1, LM9 = A3:B3:C1, CK = White fluorescent light.

Non-photochemical quenching [NPQ] increased rapidly with time under all light modes in the rocket plants. It was highest in plants grown under LM3 treatment at 40–300 seconds (Fig 9A). The photochemical quenching coefficient [qP] rapidly increased with continued light exposure at all time points. The [qP] was highest under CK at 60–100 and 160–300 seconds, while the highest values were observed under LM2 at 120–140 seconds (Fig 10A).

**Fig 11:**
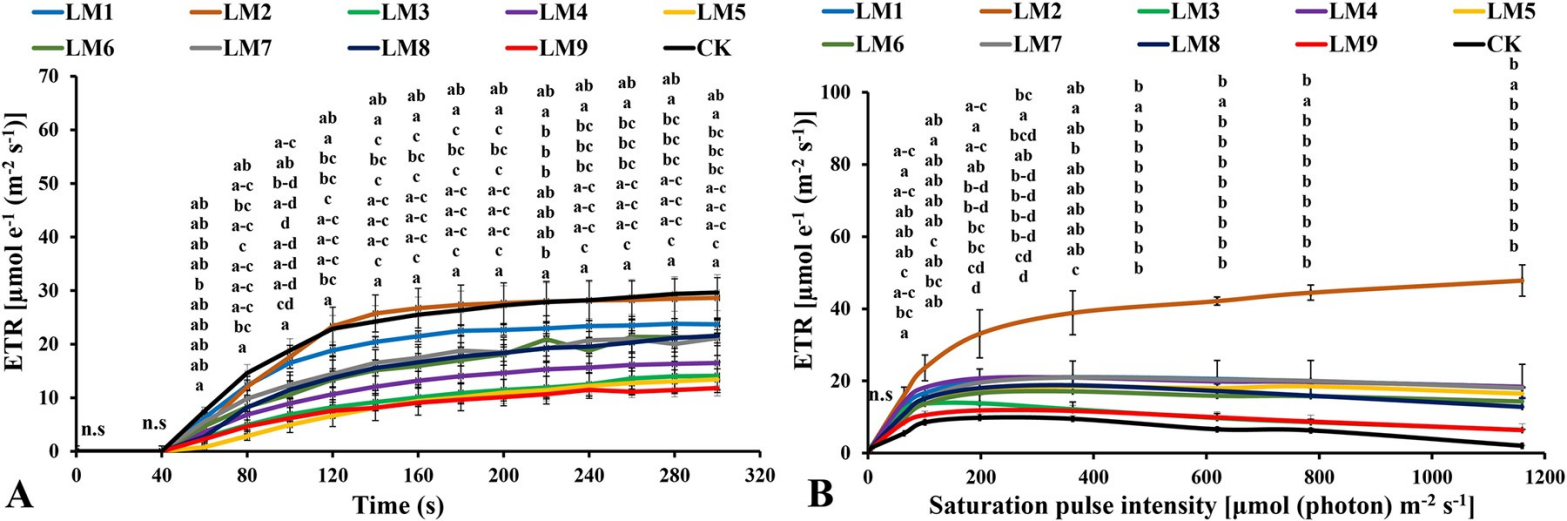
Effects of LED light modes on chlorophyll *a* fluorescence induction kinetics of the dark-acclimated (A) and RLC of the light-acclimated (B) of electron transport ratio (ETR) in rocket leaves. The points are means ± SE of 4 replicates followed by the same letters meaning no different significantly according to the Duncan test (P ≤ 0.05). Sort significance letters from top to bottom according to the treatments (ML1-CK). LM1 = A1:B1:C1, LM2 = A1:B2:C2, LM3 = A1:B3:C3, LM4 = A2:B1:C2, LM5 = A2:B2:C3, LM6 = A2:B3:C1, LM7 = A3:B1:C3, LM8 = A3:B2:C1, LM9 = A3:B3:C1, CK = White fluorescent light.

The electron transport rate [ETR] increased rapidly with an increase in light exposure at all time points under all light modes. The highest value of ETR was observed at CK at 60–100 seconds and 240–300 seconds (Fig 11A), while it was the highest under LM2 treatment at 160–220 seconds (Fig 11A).

### 3.5. Chlorophyll *a* fluorescence measurement of light-acclimated samples

The rapid light curves (RLCs) of light-acclimated photosynthetic quantum yields for PSII were measured. Photosynthetic electron transport activity was sensitive to light energy and significantly correlated with the oxidation state of the transferred electrons. The effective quantum yield of PSII photochemistry [Y(II)] gradually decreased with increasing light intensity for all light modes. The Y(II) of plants grown under LM2 light mode was significantly higher than others, at almost all light intensities (Fig 6B). The quantum yield of regulated energy dissipation of PSII [Y(NPQ)] increased rapidly with increasing light intensity in all light modes used; it was higher in plants under CK, LM6, and LM2 at 664, 101–619 and 785–1160 μmol m^-2^ s^-1^light intensities, respectively (Fig 7B). The quantum yield of unregulated energy dissipation in PSII [Y(NO)] was the highest at LM7 light mode (Fig 7B). The [Y(NO)] increased rapidly when initially exposed to light and stabilized after a small decrease in some treatments. The [Y(NO)] was higher in plants than in other treatments under LM7 and LM8 at light intensities of 0–363 and 619–1160 μmol m^-2^ s^-1^, respectively (Fig 8B).

Non-photochemical quenching [NPQ] was the highest under LM2 treatment (Fig 9B). The [NPQ] gradually increased with increasing light intensity in all treatments. The [NPQ] was significantly higher under LM6 and LM2 treatments compared to the other treatments at light intensities of 0–198 and 363–1160 μmol m^-2^ s^-1^, respectively (Fig 9B). The photochemical extinction coefficient [qP] gradually decreased with increasing light intensity under all tested light quality regimes in plants. The [qP] was significantly lower under CK compared to the other treatments at all light intensities in plants (Fig 10B), while it was the best under LM6 before the light intensity reached 6 μmol m^-2^ s^-1^and LM2 after the light intensity reached 64 to 1160 μmol m^-2^ s^-1^ (Fig 10B). The electron transport rate increased with increasing light intensity and became constant at a light intensity of 363 μmol m^-2^ s^-1^, while it decreased in some treatments after reaching 198 μmol m^-2^ s^-1^. The ETR of plants grown under LM2 was significantly higher than other treatments (Fig 11B).

On the other hand, according to the R-values, the order of influence of the three factors on chlorophyll *a* fluorescence measurements of rocket plants was observed in this study (Table 4). Table 4 shows that the order of impact of the three factors on Y(II), Y(NPQ), Y(NO), NPQ, qP, and ETR was (C > B > A), (B > C > A), (C > A > B), (C > B > A), (A > B > C), and (C > B > A), respectively.

**Table 4:** Results of the range and ANOVA of the L9 (3^3^) matrix for the influence of combined, intensities of LEDs light (A), light spectral ratios (B), and photoperiod (C) on chlorophyll *a* fluorescence measurements of rocket plants.

Parameters		Factors			ELF	BCm
		A	B	C		
**Y(II)**	*R* value	0.059	0.068	0.073	C > B > A	A_1_B_2_C_2_
	*P* value	0.3418	0.2201	0.2298		
**Y(NPQ)**	*R* value	0.058	0.105	0.060	B > C > A	A_1_B_3_C_3_
	*P* value	0.3028	0.0227	0.2538		
**Y(NO)**	*R* value	0.085	0.039	0.132	C > A > B	A_2_B_2_C_3_
	*P* value	0.2290	0.7235	0.0567		
**NPQ**	*R* value	0.153	0.214	0.287	C > B > A	A_3_B_2_C_1_
	*P* value	0.4195	0.1593	0.0482		
**qP**	*R* value	0.102	0.089	0.080	A > B > C	A_1_B_2_C_2_
	*P* value	0.2810	0.3492	0.3792		
**ETR**	*R* value	5.11	5.52	5.92	C > B > A	A_1_B_2_C_2_
	*P* value	0.2984	0.2279	0.2338		

Range value (*R*)–the range of difference between the maximum and minimum average; (*P*-value)–ANOVA analysis of variance; ELF–The most influential level factors on the parameter gradually; BCm–The best level combination for each parameter.

Based on the average of chlorophyll *a* fluorescence measurements derived from three factors at each level, the A_1_B_2_C_2_ was the best combinations gave the highest Y(II), qP, and ETR, which indicated that the maximum of these parameters presented at (intensity 220 μmol m^-2^ s^-1^ + ratio (R3:G0:B7) + photoperiod 12 h/12 h). While the best combination of different factors with the levels for the highest Y(NPQ), Y(NO), and NPQ was A_1_B_3_C_3_, A_2_B_2_C_3_, and A_3_B_2_C_1_, respectively.

ANOVA (Table 4) showed that these three factors were no significant effects on chlorophyll *a* fluorescence measurements of rocket plants (*p* < 0.05), excepted factor B on Y(NPQ) and factor C on NPQ had significant effects.

## 4. Discussion

Morphological and physiological characteristics of plants are strongly affected by the light intensity [13, 15–17, 30, 31], colors ratio [32, 33] and photoperiod [13, 20]. In this research, the effects of these 3 factors on some morphological, physiological, and biochemical traits, and plants’ photosynthetic efficiency were studied to find out the best combination of light mode/s that can be optimal for rocket plants performance.

Light absorption is affected by its intensity, wavelength, and angle of downfall [34], as well as the total leaves area. Rocket plants reacted strongly to LM1 not only in terms of plant height, but also total leaf area and root length (Table 2). Similar results were observed in spinach Nguyen, Tran [16] and in lettuce seedlings under high light intensity [20, 35]. However, da Silva, da Costa [36] and Kim and Hwang [37] reported that a combination of red and blue lights was positive for plant height, total leaves area, and root length when they grew under low light intensity.

In general, the higher light intensity is expected to result in higher biomass accumulation [38]. Lettuce plants grown under 220 μmol m^-2^ s^-1^ have increased dry weight content [15]. Rocket grown under the spectrum combination of 50% of red, 20% of green and 30% of blue colors (LM3) had the highest dry weight content. However, changes in the dry weight content observed in this study differed from those noted previously in other studies [16, 35] done on lettuce plants. These studies reported that a mixture of red and blue LED lights was the best treatment for dry matter increment. This may be due to a minor photosynthates allocation to the roots, as previously assumed [11, 15, 39].

One of the most important factors that influence photosynthesis process and primary plant production is photosynthetic pigments [40, 41]. In current study, light quality, intensity, and photoperiod significantly affected the chlorophyll content of rocket plants. The mixture of red and blue LED light LM4 (R7:G0:B3) was favorable for Chl *a*, but LM3 (mixture of red, green, and blue LED light- R5:G2:B3) was the best for Chl *b*, while LM1 (R7:G0:B3) was the best for carotenoid. These results are consistent with those noted previously [12, 16, 35, 42, 43], which showed that Chl *a*, *b*, and carotenoid contents were higher under a mixture of red and blue LED light as compared to white fluorescent light. Often a mixture of red and blue light with a high red to blue light ratio increases the chlorophyll contents in the leaves of the plant due to the activation of the formation of chloroplasts. Krzeszowiec and co-workers [44] reported that powerful blue light caused chloroplasts to aggregate at cell walls parallel to the light path (avoidance response), while weak blue light caused them to relocate to the most illuminated cell walls (accumulation response).

Current study demonstrated the importance of red light in the accumulation of some biochemical compounds in rocket *E*. *Sativa*. LM4 was beneficial to increase soluble sugar and soluble protein levels. Similarly, Bian, Cheng [45] showed that soluble sugar levels and soluble proteins were higher in lettuce under continuing red, green, and blue (4:1:1) LEDs illumination. However, Xiaoying, Shirong [46] showed that soluble sugar levels were higher in tomato seedlings under blue light, while Cui, Ma [47] showed that soluble sugar levels were higher in pepper, cucumber, and tomato seedlings under red and red + blue lights. These results indicated that soluble sugars and proteins respond to the light quality in vegetable crops.

LM3 (ratio R5:G2:B3 + intensity 220 ± 2 μmol m^-2^ s^-1^ + photoperiod 14 h/10 h) was of beneficial to rising Vitamin C concentration followed by LM6 (ratio R5:G2:B3 + intensity 190 ± 2 μmol m^-2^ s^-1^ + photoperiod 10 h/10 h), LM7 (ratio R7:G:B3 + intensity 160 ± 2 μmol m^-2^ s^-1^ + photoperiod 14 h/10 h), and LM9 (ratio R5:G2:B3 + intensity 160 ± 2 μmol m^-2^ s^-1^ + photoperiod 12 h/12 h) treatments. The results indicated that the accumulation of vitamin C is affected by the ratio of LED light with a high red ratio, regardless of the optical intensity or photoperiod. This finding contradicted with the results shown by Lin, Huang [48], where the accumulation of vitamin C was affected by the quality of the light with a higher percentage of blue LED light on the lettuce plant.

In the present study, it was observed that under intensity (190 ± 2 μmol m^-2^ s^-1^) and photoperiod (14 h), a mixture of R3:B7 LED light was more effective in increasing nitrate concentrations than other treatments. These results were supported by the study of Bian, Cheng [45] and Yousef, Xu [43], which showed that the mixture of red and blue LED light was more effective in increasing nitrate concentrations in hydroponically grown lettuce.

The results indicated that different light intensities, qualities, and photoperiods of LED specifically regulated energy allocation, heat dissipation, cyclic electron transport, and the activity of the photosynthetic electron transport chain [49]. These factors can indirectly impact photosynthetic electron transport by adjusting multiple biochemical processes like endogenous hormonal balance and metabolic reactions and directly impact the photochemical reaction during short-term lighting [50].

In this experiment, when plants were dark-acclimated, Y(II) increased continuously over time (Fig 6A), while Y(II) decreased in the light-acclimated plants with the increase of PAR from 0 to 1160 μmol m^-2^ s^-1^. In the latter case, the reaction centers were transiently inactive, and all-electron acceptors were fully reduced, thus [Y(II)] decreased when the intensity of saturated light increased (Fig 6B). The results showed that after plants dark-acclimation the best light mode that squandered excess lowering energy were LM2 and CK (Fig 6A), whereas the best treatment squandered excess lowering energy was LM2 after light-acclimation (Fig 6B). The results obtained differed from those reported previously other researches [16, 50–52], where it was reported that the mixture of red and blue light with a high ratio of red to blue led to an increase of Y(II)max (Fv/Fm).

The Y(NPQ) corresponds to the fraction of energy dissipated in the form of heat by the regulated photoprotective mechanism. The high values of Y (NPQ) are a sign of high cooling capacity when the value of Y(II) is near zero at a high quantum flux density [53]. It was noted in this study that the best treatments that allowed the plants to have the ability to protect themselves from the damages caused by excess lighting and increased their high absorptive capacity in dark-acclimated were observed under LM3 (Fig 7A), but under LM2 and LM6 in the light-acclimated plant (Fig 7B). This was in agreement with the findings of Yang, Xu [50] in tomato seedlings.

The Y(NO) parameter is an indicator of the negative energy portion that dissipates in heat and fluorescence, mainly due to closed PSII reaction centers. The high values of [Y(NO)] reflect plants’ inability to protect themselves from excessive light damage [53]. The Y(NO) decreased slowly in both dark and light-acclimated plants. It was noted that the best treatments for increasing plants’ ability to protect themselves from damage were the LM2 (Fig 8A and 8B), whether under dark-acclimated or light-acclimated, while the other treatments differed the plants’ ability to protect themselves from damage under dark or light acclimated. This may be due to the difference in light qualities or light intensities, or photoperiods [50].

In order to reduce photo-damage, plants have developed several protective mechanisms, including non-photochemical cooling [NPQ] that quenches the agitation of the chlorophyll inside the light-harvesting antenna of PSII by converting excitation energy into thermal energy, which can then be sent out [54, 55]. There may be cooperation or complementarity between photosynthetic respiratory metabolism and NPQ in order to maintain oxidative balance within cells. Some other photoprotective mechanisms closely related to NPQ also have been identified, such as the water-water cycle and periodic electron transport and in regulating NPQ induction [56–58]. The value of NPQ increased continuously with increasing time or light intensity under both dark and light acclimation conditions. The best combinations were LM8 and LM3 after dark-acclimation (Fig 9A), while the best combinations were LM2 and LM6 after plants’ light-acclimation (Fig 9B). The photosynthetic machinery of plants adapted to dark or light differed in dissipating the extra energy that may cause damage to plant tissues; the LM6 combination was the best to dissipate the extra energy and converting excitation energy into thermal energy. These findings can be supported by Hoffmann, Noga [59], He, Qin [60], and by Avercheva, Berkovich [51].

Under light-acclimation conditions, the photochemical quenching coefficient (qP) decreased with an increase in PAR (0, 2, 6, 64, 101, 198, 363, 619, 785 and 1160 μmol m^-2^ s^-1^) in rocket leaves but increased with time (40–300 s) (Fig 10A and 10B). The best light mode that assisted in maintaining higher number of open reaction centers were CK and LM2 in dark-acclimated plants (Fig 10A), while the best light mode that supported maintaining, as much as possible, the largest possible number of open reaction centers were LM2 and LM7 in the light-acclimated plants (Fig 10B). These results are consistent with those noted previously by other studies [50, 51, 60], where a mixture of red and blue light increased the qP.

The electron transport ratio (ETR) values were achieved by adopting the procedure of Schreiber [61]. The best light combinations that increased electron transport rate were CK and LM2 in dark-acclimated plants (Fig 11A), while the best treatment that increased electron transport rate in light-acclimated plants was only LM2 (Fig 11B). The results differed with those noted previously by other investigations [43, 59, 60], where they have reported that a mixture of red and blue light with a high ratio of red to blue increased the ETR. Liu and van Iersel [62] reported that the green spectrum alone (G100), or blue with green (B20:G80), or red with green (R20:G80 or R80:G20), or a mixture of red, green, and blue (R64:G20:B16), had the strongest effect of the electron transport rate (ETR) in lettuce “Green Towers” plants.

## 5. Conclusion

Artificial light is very important for countries that do not have natural sunlight, especially LED light, because it is less in consuming electricity, lower in temperature, and longer in a lifetime. In this research, the effects of different combinations of light intensities and qualities with different photoperiods on plant growth parameters and photosynthetic performance were studied. The best results were achieved under a mixture of red and blue light and a photoperiod less than 14 hours per day. The variation among the studied characteristics (lack of clear trends) suggests that analyzing the linkage between the studied morphological, biochemical, and physiological characteristics is insufficient to understand the effect of combination of light intensity, its quality, and photoperiod) on plant growth, it is necessary to study more features such as photosynthesis intensity and molecular analysis to understand such interactions better.

Moreover, the obtained results in this work disagree with our previous study conducted on cucumber plants (publication is under reviewing process); thus, it has been confirmed that the response of plants to different light modes (regimes) is species dependent. This response will also vary from one to another genotype of the same spices. Thus, the latter issue will be the core of our next investigations.
